# Challenges in Exploiting Human H Ferritin Nanoparticles for Drug Delivery: Navigating Physiological Constraints

**DOI:** 10.1002/wnan.2016

**Published:** 2024-11-14

**Authors:** Alberto Macone, Chiara Cappelletti, Alessio Incocciati, Roberta Piacentini, Sofia Botta, Alberto Boffi, Alessandra Bonamore

**Affiliations:** ^1^ Department of Biochemical Sciences "Alessandro Rossi Fanelli" Sapienza University of Rome Rome Italy

**Keywords:** biodistribution, cell trafficking, drug delivery systems, ferritin, protein nanoparticles

## Abstract

Over the past two decades, ferritin has emerged as a promising nanoparticle for drug delivery, catalyzing the development of numerous prototypes capable of encapsulating a wide array of therapeutic agents. These ferritin‐based nanoparticles exhibit selectivity for various molecular targets and are distinguished by their potential biocompatibility, unique symmetrical structure, and highly controlled size. The hollow interior of ferritin nanoparticles allows for efficient encapsulation of diverse therapeutic agents, enhancing their delivery and effectiveness. Despite these promising features, the anticipated clinical advancements have yet to be fully realized. As a physiological protein with a central role in both health and disease, ferritin can exert unexpected effects on physiology when employed as a drug delivery system. Many studies have not thoroughly evaluated the pharmacokinetic properties of the ferritin protein shell when administered in vivo, overlooking crucial aspects such as biodistribution, clearance, cellular trafficking, and immune response. Addressing these challenges is crucial for achieving the desired transition from bench to bedside. Biodistribution studies need to account for ferritin's natural accumulation in specific organs (liver, spleen, and kidneys), which may lead to off‐target effects. Moreover, the mechanisms of clearance and cellular trafficking must be elucidated to optimize the delivery and reduce potential toxicity of ferritin nanoparticles. Additionally, understanding the immune response elicited by exogenous ferritin is essential to mitigate adverse reactions and enhance therapeutic efficacy. A comprehensive understanding of these physiological constraints, along with innovative solutions, is essential to fully realize the therapeutic potential of ferritin nanoparticles paving the way for their successful clinical translation.

## Graphical abstract

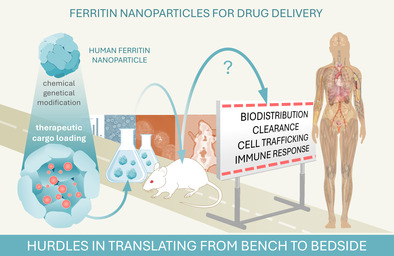

Challenges in Advancing Ferritin Nanoparticles from Laboratory Research to Clinical Use. Ferritin nanoparticles show promising potential in targeted drug delivery, but several key challenges must be addressed before they can reach clinical application. These challenges include optimizing chemical and genetic modifications to enhance nanoparticle stability, achieving efficient and specific drug loading, and targeting therapeutic payloads precisely to disease sites. Additionally, understanding complex in vivo behaviors—such as biodistribution, clearance rates, cellular trafficking, and immune interactions—is critical to ensure safety and efficacy. Addressing these challenges will be of paramount importance in advancing ferritin‐based nanomedicine from preclinical research to practical and safe applications for patients.

**Abbreviations**
CD63: cluster of differentiation 63
CD71: cluster of differentiation 71 or transferrin receptor 1
CXCR4: C‐X‐C motif chemokine receptor 4
EGF: epidermal growth factor
EPR: enhanced permeability and retention
EV: extracellular vesicle
IL4R: interleukine 4 receptor
MARCO: macrophage receptor with collagenous structure
Msr1: macrophage scavenger receptor 1
NCOA4: nuclear receptor activator 4
NF‐kB: nuclear factor kappa‐light‐chain‐enhancer of activated B cells
NRP‐1: neuropilin‐1 protein
PAS: proline‐alanine‐serine
PASE: proline‐alanine‐serine‐glutamate
PEG: polyethylene glycol
RGD: arginine‐glycine‐aspartate
RGDK: arginine‐glycine‐aspartate‐lysine
RGE: arginine, glycine, glutamate
SCARA(1–5): Scavenger receptor class A member 1–5
Tim(1–2): T‐cell immunoglobulin mucin receptor 1 and 2
TRAIL: TNF‐related apoptosis‐inducing ligand
XTEN: unstructured hydrophilic biodegradable polypeptide

## 1 Introduction

### 1.1 Ferritin Physiological Function

**FIGURE 1:**
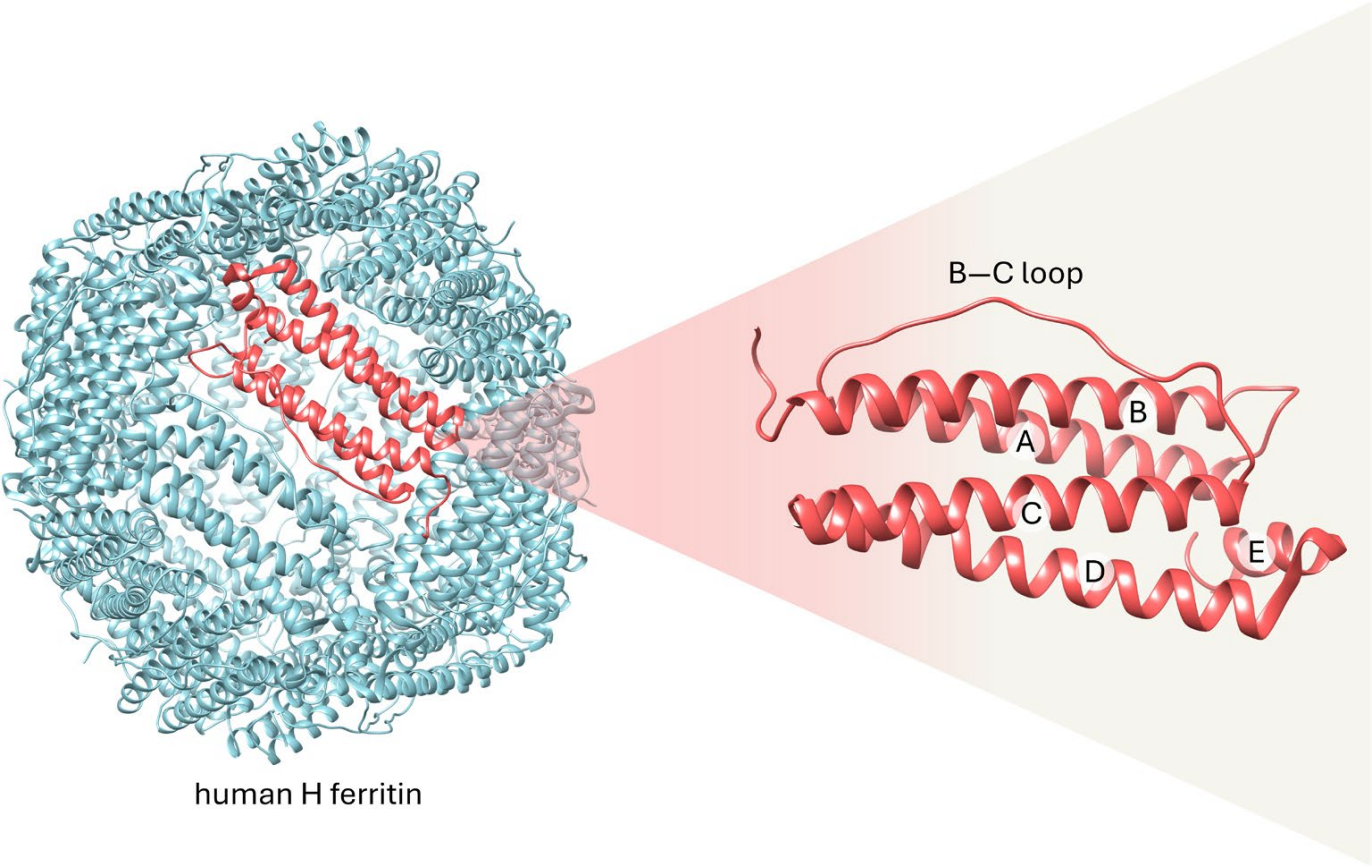
Structure of human H ferritin (PDB entry 2FHA). Human H ferritin nanoparticle is made up of 24 identical subunits that self‐assemble in octahedral (432) symmetry, with an outer diameter of 12 nm and an inner diameter of 8 nm. Each subunit consists of four antiparallel alpha helices (from A to D) plus a short helix E that extends toward the inner cavity. Helices B and C are connected by a long unstructured loop, which is exposed on the outer surface of ferritin. This loop is crucial for the recognition of the transferrin receptor 1 (CD71).

Ferritin is a highly conserved protein essential for iron storage and regulation, playing a pivotal role in iron homeostasis and cellular protection against oxidative stress. Structurally, ferritin is a complex protein assembly organized into a spherical shell composed of 24 subunits (Crichton 1973). Each ferritin molecule consists of two types of subunits: heavy (H) and light (L) chains, which cooperatively form the characteristic nanocage structure (Boyd et al. 1985; Figure 1).

The ratio of H to L subunits in ferritin is dynamically regulated depending on the specific cell type and physiological context. For example, tissues such as the liver and spleen, which are primary sites for iron storage, exhibit a higher concentration of L ferritin due to its structural stability and efficiency in iron storage. In contrast, tissues such as the heart, brain, and kidneys, which are involved in high iron oxidation activity, predominantly contain H ferritin. This is because H ferritin possesses a ferroxidase center that facilitates the rapid oxidation of Fe^2+^ to Fe^3+^ in conjunction with O_2_ reduction (Levi et al. 1992). The ferritin nanocage features a central cavity where iron ions are safely stored in a bioavailable form. The structural integrity of ferritin is crucial for its function in iron sequestration and release. One of ferritin's primary roles is to act as an intracellular iron reservoir, buffering fluctuations in iron availability and preventing iron toxicity (Arosio and Levi 2002). Within the nanocage, excess iron is stored as ferric ions (Fe^3+^), which are insoluble and less reactive than ferrous ions (Fe^2+^). This sequestration mitigates oxidative damage caused by free radicals generated through Fenton chemistry, thereby safeguarding cellular integrity and function (Aust 1995).

Ferritin also plays a pivotal role in regulating systemic iron levels by dynamically releasing stored iron in response to cellular demands. Various physiological cues, including iron deficiency, hypoxia, and oxidative stress, trigger the controlled release of iron from ferritin. This process involves the enzymatic degradation of ferritin and the subsequent mobilization of the stored iron for utilization in essential metabolic pathways, such as heme synthesis, DNA replication, and mitochondrial respiration (Kidane, Sauble, and Linder 2006).

Beyond its intracellular roles, ferritin is also present in plasma, albeit at much lower concentrations. Plasma ferritin lacks a direct storage function and serves as a clinical biomarker for body iron storage and various pathological conditions (Wang et al. 2010).

Emerging evidence suggests that ferritin exerts pleiotropic effects beyond its canonical role in iron metabolism. Ferritin and its subunits are involved in cellular signaling pathways that regulate inflammation, immune response modulation, and cell proliferation (Kernan and Carcillo 2017; Moreira, Mesquita, and Gomes 2020; Alsyamy et al. 2022). Additionally, ferritin exhibits immunomodulatory properties, functioning in both pro‐inflammatory and anti‐inflammatory responses depending on its subcellular localization and context (Gehrer et al. 2023; Ruscitti et al. 2020). These immunoregulatory roles highlight the complex interplay between ferritin and the immune system, essential for maintaining homeostasis and mounting effective immune responses.

Thus, beyond its role as an iron storage protein, the complex physiology of the ferritin molecule must be carefully considered when developing ferritin‐based nanoparticles for biomedical applications. These applications primarily include imaging and targeted drug delivery, exploiting ferritin's capacity to encapsulate and release various therapeutic agents in a controlled manner. Understanding and manipulating these multifaceted roles of ferritin are crucial for advancing its use in nanomedicine.

### 1.2 Ferritin and Biomedical Applications

In the last decade, ferritin has gained considerable attention in biomedical research due to its nanoarchitecture and versatile functionality. Its structural framework offers immense potential for drug delivery applications (Tu et al. 2023), imaging (Ruggiero et al. 2019), vaccine development (Weidenbacher et al. 2023; Vu et al. 2023), and as a scaffold for presenting therapeutic peptides (Lee et al. 2020; Parisi et al. 2024; Incocciati et al. 2024).

**FIGURE 2:**
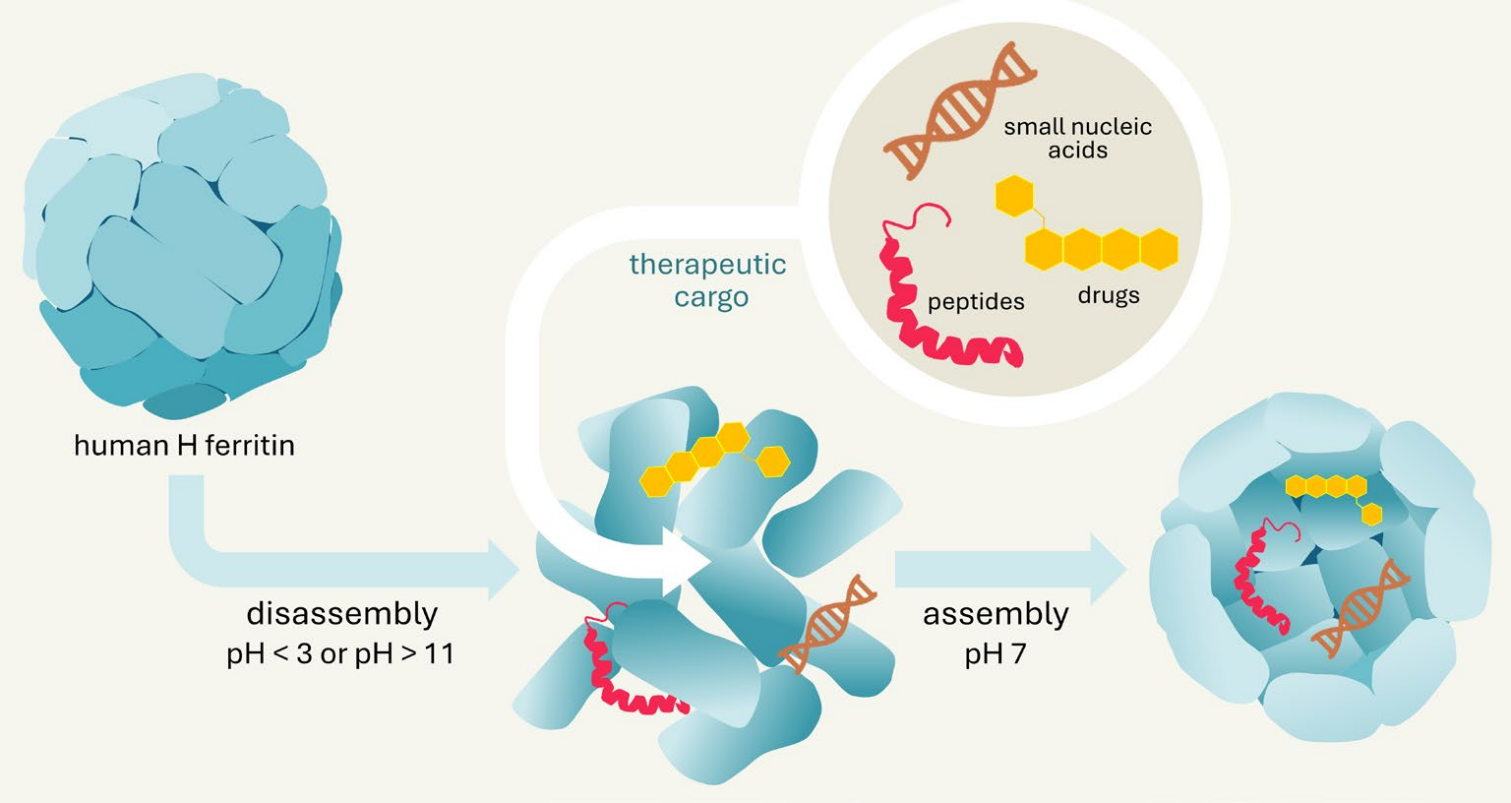
Human H ferritin can be disassembled and reassembled by changing the pH. At pH values lower than 3 or higher than 11, ferritin completely disassembles. Reassembly of the nanocage occurs when the pH is restored to neutral. During this reassembly stage, therapeutic molecules or imaging agents can be added and subsequently encapsulated within the protein shell.

Ferritin's unique structure, characterized by its biocompatible nature and hollow interior, provides an attractive platform for encapsulating and delivering therapeutic agents including conventional drugs (Chen et al. 2022; Jáklová et al. 2021; Sitia et al. 2020; Wu et al. 2022) contrast agents (Calisti et al. 2018; Affatigato et al. 2023), therapeutic proteins (Macone et al. 2019), and small interfering RNA (Yuan et al. 2022). Indeed, the ferritin shell can disassemble and reassemble in response to pH changes (Yousefi et al. 2024), stably incorporating the selected cargo (Kim et al. 2011; Figure 2). Targeted delivery and imaging exploit the ability of heavy chain ferritin (H ferritin) to bind the transferrin receptor CD71, which is overexpressed in metabolically active iron‐demanding cells including tumor cells (Montemiglio et al. 2019). Additionally, ferritin can be functionalized on both internal and external surfaces to modulate cargo capacity (Incocciati et al. 2023; Z. Wang, Zhao, et al. 2022) and recognize specific molecular targets other CD71 on the cell surface (Khoshnejad et al. 2018; Deng et al. 2023).

Beyond conventional drug delivery, ferritin has emerged as a promising platform for vaccine development and as a scaffold to present peptides. By engineering ferritin to display antigenic peptides or proteins on its surface, it is possible to mimic the structural features of pathogens, eliciting potent immune responses and offering a novel approach to vaccination strategies (Weidenbacher et al. 2023; Ober Shepherd et al. 2024).

This dual functionality of ferritin highlights its versatility and potential as a multifaceted tool in biomedicine.

## 2 Challenges of Using Ferritin as a Nanovector

The transition of ferritin from its physiological role in iron storage to a tailored drug delivery system presents many challenges. To fully exploit its potential, a comprehensive understanding of ferritin's physiological functions, interactions within biological systems, and the impact of these factors on its efficacy as a drug carrier is essential.

In light of these considerations, this review will delve into the challenges surrounding the utilization of ferritin as a nanoparticle for drug delivery. Although ferritin has been studied for at least 20 years in this context, the transition from bench to bedside remains largely unfulfilled. This ongoing gap highlights the need to overcome the hurdles currently hindering clinical translation. By exploring the physiological challenges and complexities of ferritin‐based drug delivery systems, we aim to offer insights that will pave the way for more effective and clinically viable therapeutic solutions.

### 2.1 Biodistribution

To fully understand a nanoparticle's pharmacology, comprehensive testing in preclinical models is crucial, including toxicology, pharmacokinetics, and efficacy assessments. Quantitative biodistribution studies are essential to measure the total number of nanoparticles in the body at specific time points after injection or exposure. Also in the case of human ferritin, studying its biodistribution is crucial for its application in biomedical fields. A major challenge in translating ferritin nanoparticles to clinical settings is the detailed understanding of their behavior post‐administration in the human body. This is especially important since ferritin is also an endogenous protein with specific physiological functions.

One of the key challenges is that most studies are conducted in mice, using different types of ferritin—ranging from equine to human—for various applications like drug delivery and imaging. This diversity makes it particularly difficult to derive insights that can effectively support the translation of research from the laboratory to clinical practice.

Ferritin, as an endogenous protein, is found not only within cells but also circulating in bodily fluids such as serum and plasma (Garcia‐Casal et al. 2021). In the bloodstream, ferritin can exist in various forms, including free ferritin, ferritin‐bound iron, and ferritin‐containing exosomes (Palsa et al. 2023; Yanatori, Kishi, et al. 2023; Yanatori, Nishina, et al. 2023; Ghosh, Hevi, and Chuck 2004). Its biodistribution shows notable tissue specificity, varying across different organs and cell types, overlapping the distribution of transferrin receptor 1 (TfR1 or CD71), which mediates ferritin uptake (Montemiglio et al. 2019). High CD71 expression is typically found in tissues with high metabolic activity and iron demand, such as the liver, spleen, bone marrow, and reticuloendothelial system (Das et al. 2022; Fillebeen et al. 2019). Factors such as cellular turnover, iron status, inflammatory responses, and pathological conditions also influence the biodistribution of endogenous ferritin (Kernan and Carcillo 2017; Fonseca et al. 2023). Conversely, little or nothing is known about how human ferritin biodistributes in humans when administered as a biopharmaceutical.

To the best of our knowledge, the first biodistribution data available for human H‐ferritin without any surface modifications were obtained in studies where it was used as an electron‐dense label for electron microscopy and later as a superparamagnetic contrast agent for MRI in murine models. These studies revealed that human H‐ferritin has a blood clearance time of only a few minutes and is primarily taken up by the reticuloendothelial system and the lungs. Two hours after injection, the highest concentrations were found in the liver, spleen, and lungs (Liang et al. 2018; Huang et al. 2021). Similar biodistribution patterns have been reported in studies involving nonhuman ferritins, such as horse spleen ferritin. For instance, a study found that magnetoferritin was quickly taken up by the liver, spleen, and lymph nodes in mice, with no differences in biodistribution observed when apoferritin was injected (Bulte et al. 1995).

Other studies using horse spleen ferritin show that its biodistribution can change significantly when positive charges are introduced on its surface (cationized ferritin). Specifically, superparamagnetic cationized ferritin showed a high affinity for the renal glomerulus, the extracellular matrix of hepatic sinusoids, the glycocalyx of alveolar epithelial cells, and macrophages in the spleen (Bennett et al. 2008; Bulte et al. 1995). Furthermore, its biodistribution shifts under pathological conditions such as glomerulosclerosis, atherosclerosis, and tumors (Beeman et al. 2013). A similar scenario could potentially occur with modifications to human H‐ferritin, altering its biodistribution and targeting properties.

In numerous pathological states, particularly tumors, cellular iron demand is markedly elevated, positioning human H ferritin as a key player. In targeting tumor cells, ferritin exploits its intrinsic ability to bind to the CD71 receptor, which is overexpressed in iron‐demanding tumor cells (Shen et al. 2018). Besides receptor‐mediated cellular uptake, ferritin nanoparticles also offer passive targeting via the enhanced permeability and retention (EPR) effect (Zhu et al. 2021). Ferritin's uniform size of 12 nm is ideal for exploiting the EPR effect caused by the disordered blood and lymphatic vessels in tumor tissues. Unlike clinically approved nanoparticle drug delivery systems such as liposomal doxorubicin (Doxil) and albumin‐paclitaxel (Abraxane), which rely solely on the EPR effect (Barenholz 2012; Gradishar 2006), ferritin nanoparticles employ a dual targeting strategy. By combining CD71 receptor binding with the EPR effect, ferritin nanoparticles enhance tumor localization and reduce off‐target effects. However, when using ferritin for theranostic purposes, potential off‐target effects must be considered. As an acute‐phase protein, ferritin can induce pro‐inflammatory responses, a critical aspect often overlooked in studies utilizing ferritin nanoparticles for drug delivery. A study in mice demonstrated that administering human apoferritin at 60 μg/g body weight, a dose comparable to those used in delivery experiments, leads to systemic and hepatic inflammation with increased neutrophil infiltration, indicative of hyperferritinemic syndrome (Jia et al. 2022). This inflammation is mediated by the macrophage scavenger receptor 1 (Msr1) on neutrophils, which binds ferritin and initiates inflammatory signaling. Additionally, ferritin's ability to cross the blood–brain barrier, its presence in serum and cerebrospinal fluid, and the existence of ferritin receptors in nerve cells suggest that exogenous ferritin nanoparticles could have also neurological consequences as they modulate glutamate homeostasis independently of iron (Krisanova et al. 2014; Porras and Rouault 2022). These potential effects must be carefully evaluated when using exogenous human ferritin for biomedical applications.

Our understanding of the physiological impact of ferritin nanoparticles on humans is constrained because existing data rely solely on murine models. In mice, Tim2 serves as the H ferritin receptor, but humans lack Tim2 orthologs and rely on CD71 instead (Han et al. 2011; Li et al. 2010). This difference poses a significant challenge in translating findings from murine studies to humans. Furthermore, CD71 expression levels vary among different tumors and stages of tumor progression (Candelaria et al. 2021). For instance, high CD71 expression is found in head and neck, colorectal, and cervical cancers, but not in carcinoid, prostate, or testicular tumors (Shen et al. 2018). CD71 is also ubiquitously expressed in healthy tissues, including bone marrow, lung, colon, and liver, risking undesired drug accumulation. Therefore, enhancing active targeting through chemical or genetic modifications is often necessary to optimize biodistribution toward tumor cells. For ferritin nanoparticles, this has been achieved by genetically engineering human H ferritin to display specific sequences. Recent studies highlighting improved tumor targeting through genetic modifications, alongside biodistribution data are reported in Table 1. One approach was developing an integrin α2β1‐targeting H ferritin for glioma treatment, that showed enhanced doxorubicin loading capacity and tumor targeting capability after crossing the blood–brain barrier (Huang et al. 2021; Table 1). Although this nanoparticle significantly suppressed tumor progression and extended survival in glioma mouse models, its biodistribution was not confined to the tumor, but also involved healthy tissues, including the liver and kidneys.

**TABLE 1:** Human H ferritin engineering to improve biodistribution through tumor targeting.

Ferritin surface modification	Cellular target	Tumor	Encapsulated drug	References
Integrin α2β1 targeting peptide	Integrin α2β1	Subcutaneous and orthotopic glioma	Doxorubicin	Huang et al. 2021
RGD‐derived peptide	Integrin αvβ3	Subcutaneous glyobastoma	Doxorubicin	Zhen et al. 2013
RGE‐derived peptide	NRP‐1	Intracranial glioma	STING agonist (SR717)	Wang, Tang, et al. 2022
TRAIL/IL4R binding peptide	TRAIL receptors	Pancreatic ductal adenocarcinome	—	Yoo et al. 2020
RGD peptides	Integrin αvβ3	Glyoma	Epirubicin and camptothecin	Wang et al. 2021
PAS and RGDK peptides	Integrin αvβ3/5 and neuropilin‐1	Mammary adenocarcinoma	Doxorubicin	Yin et al. 2021
PAS/PASE shielding sequence	CD71	Pancreatic ductal adenocarcinoma	Doxorubicin and mitoxantrone	Falvo et al. 2018
Epidermal growth factor	EGF receptor	Mammary adenocarcinoma	—	Li et al. 2012

Another example is the three‐amino acid sequence RGD, which targets integrin αvβ3, an angiogenesis biomarker upregulated in many tumor types (Zhen et al. 2013). In this case, the RGD‐modified nanocage loaded with doxorubicin targeted the tumor, but also accumulated in the liver.

**FIGURE 3:**
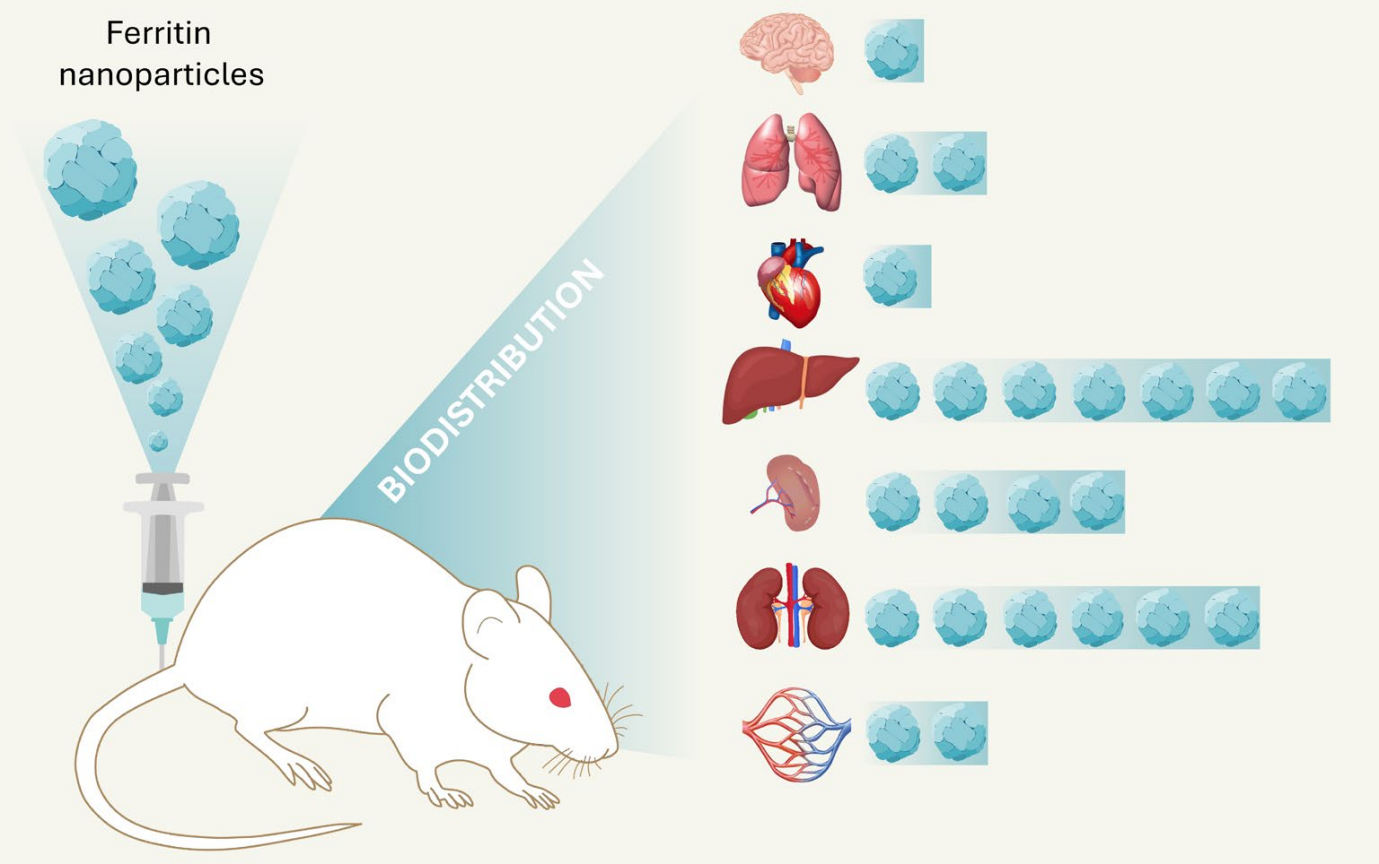
Biodistribution of ferritin nanoparticles in murine models. Most studies on the biodistribution of human H ferritin nanoparticles have been conducted using murine model systems, even though mice lack the CD71 receptor, which is involved in the recognition and uptake of H ferritin in humans. These studies employed nanoparticles designed to target tumor sites (see Table 1), loaded with specific cargoes. Direct comparison among these studies is challenging, and definitive data on biodistribution cannot be obtained. However, it is evident that ferritin predominantly accumulates in the liver, spleen, and kidneys, organs that play a major role in the clearance of ferritin.

In addition to these examples, many other studies have demonstrated that surface modifications could successfully achieve higher concentrations of nanoparticles at the tumor site. However, biodistribution remained broad, involving various organs (Wang, Tang, et al. 2022; Yao et al. 2020; Yoo et al. 2020; Wang et al. 2021; Yin et al. 2021; Falvo et al. 2018; Li et al. 2012; Figure 3).

Moreover, while numerous studies have preclinically evaluated the biodistribution of ferritin nanoparticles, most have concentrated on the pharmacokinetics of the drugs encapsulated within the nanoparticles rather than the pharmacokinetics of the ferritin cages themselves. There is a crucial need for more comprehensive measurement and evaluation in this area. To understand how various nanoparticle properties (such as size and charge) influence pharmacokinetics, it is essential to map in vivo biodistribution patterns of the human H ferritin shell, both in its native form and after modification (Kumar et al. 2023).

### 2.2 Clearance

A comprehensive grasp of ferritin clearance mechanisms is critical for refining nanoparticle design and distribution profiles. Ferritin nanoparticles primarily exit circulation through the reticuloendothelial system, particularly via uptake by liver and spleen macrophages. Factors like particle size, surface charge, and surface chemistry significantly influence the kinetics of clearance and tissue distribution (Lu et al. 2024). By customizing these properties, we can improve tissue‐specific targeting and evade immune recognition. Therefore, modifying the surface of ferritin nanoparticles can enhance their pharmacokinetic profile and therapeutic effectiveness. Typically, when nanoparticles are employed as drug delivery systems, less than 1% of the injected dose reaches the target site due to body clearance mechanisms and biological barriers. The remaining 99% often accumulates in healthy organs and tissues, potentially causing side effects (Wilhelm et al. 2016). Therefore, designing nanoparticles to achieve efficient clearance is crucial to reduce toxicity. However, balancing rapid clearance, which can diminish therapeutic effectiveness, with prolonged circulation, which can increase toxicity, is essential for developing safe and effective nanomedicines. Human H ferritin nanoparticles, with their 12 nm diameter, benefit from both CD71 recognition and the EPR effect (Li et al. 2010; Zhu et al. 2021). These dimensions also allow for relatively rapid renal clearance, reducing potential toxicity from prolonged circulation. However, the short circulation time can limit the use of ferritin as a drug delivery system. Thus, achieving a balance between these factors through specific design strategies is critical for therapeutic efficacy.

While it is known that ferritin is present in urine and correlates with serum levels (Gerday et al. 2021), the clearance of ferritin supplied as nanoformulation is not well‐documented. A study from 1982 is the only known research where serum ferritin purified from patients with hemochromatosis was labeled with ^131^I and injected into healthy adults. This study found that ferritin clearance was relatively slow, with 50% of ^131^I‐ferritin remaining in plasma at 27–30 h. In addition, this study reported that glycosylated ferritin was cleared more slowly than the non‐glycosylated fraction (Worwood et al. 1982).

All the other data on ferritin clearance come from animal studies, which indicate a half‐life of several tens of hours. For instance, a study on mice showed efficient clearance of doxorubicin‐loaded human H ferritin nanoparticles, with about 70% clearance in healthy mice, minimizing exposure of healthy organs to doxorubicin. In HT‐29 human colorectal adenocarcinoma‐bearing mice, approximately 55% of injected ferritin/doxorubicin was cleared within 96 h, indicating specific tumor accumulation (Liang et al. 2014).

**FIGURE 4:**
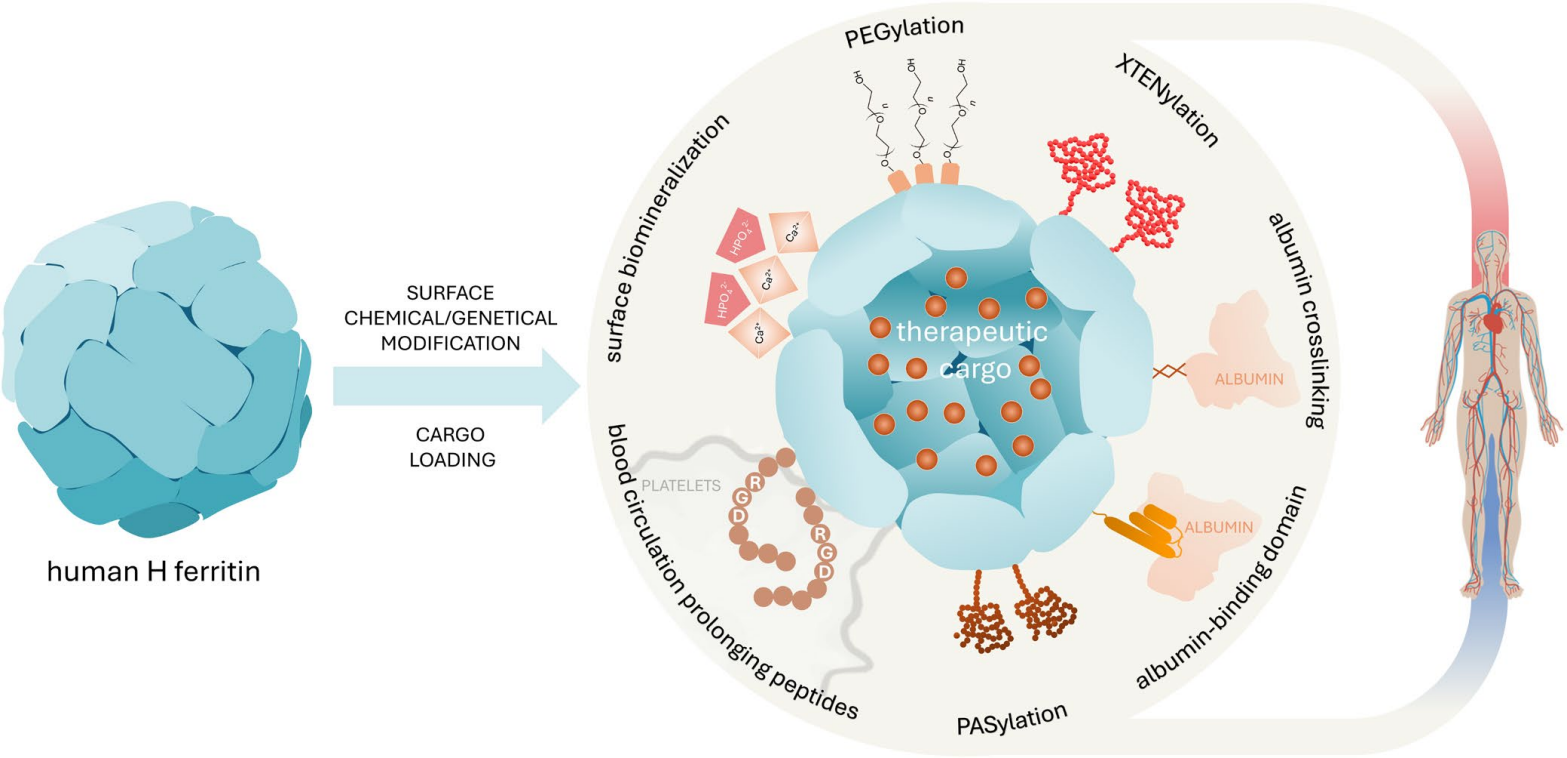
Surface modification of ferritin nanoparticles to prolong their blood circulation. Ferritin nanoparticles typically have a short half‐life in circulation. To enhance treatment effectiveness by extending their half‐life, ferritins can be modified on their external surface using both chemical and genetic methods. Chemical modifications include biomineralization, utilizing ferritin's negative surface charges as nucleation sites for calcium phosphate crystallization; PEGylation, involving the attachment of polyethylene glycol (PEG) molecules to ferritin; and crosslinking, enabling the covalent binding of albumin. Genetic modifications can include the addition of specific amino acid sequences, such as PAS, XTEN, albumin‐binding domains, and RGD sequences, to the N‐terminus. These modifications can increase the size of the nanoparticles and/or reduce off‐target uptake, thereby decreasing renal clearance and promoting longer circulation times.

Multiple studies indicate that surface modifications can extend nanoparticle half‐life (Figure 4). These modifications can be achieved through either chemical or genetic approaches. One chemical strategy involves employing biocompatible polymers such as polyethylene glycol (PEG). PEGylation improves systemic circulation time and decreases immunogenicity (Suk et al. 2016), but it may interfere with ferritin's ability to recognize CD71 and target cancer cells (Vannucci et al. 2012). On the other hand, PEGylation can be useful when ferritin is modified to bind targets other than CD71.

Another chemical modification that increases human H ferritin circulating half‐life involves surface biomineralization with calcium phosphate. This coating prevents interaction between ferritin nanoparticles and CD71, particularly at the liver level. It dissolves in the acidic tumor microenvironment, facilitating specific uptake by tumor cells (Wang, Wang, et al. 2022). Crosslinking ferritin with albumin can also reduce clearance and increase drug binding capacity (Xue et al. 2022). In addition to chemical modifications, human H ferritin has been genetically modified with half‐life extension peptides such as PAS (Yin et al. 2021; Liang et al. 2014), XTEN (Podust et al. 2016; Lee et al. 2017), albumin binding domains (Wang et al. 2018), and blood‐circulation prolonging peptides (Jin et al. 2019). PAS, a sequence rich in proline, alanine, and serine, expands hydrodynamic volumes and retards kidney filtration prolonging pharmacokinetics in vivo. XTEN, consisting of six repeating hydrophilic amino acids (alanine, glutamate, glycine, proline, serine, and threonine), offers high biodegradability and bioavailability without significant immunogenicity. Albumin‐binding domains increase circulation time by binding to human serum albumin. Blood‐circulation prolonging peptides, obtained by phage display and containing the RGD motif, enhance blood retention by binding to platelets. In addition to the strategies aimed at extending the circulating half‐life, enhancing active uptake via peptides that target specific receptors could reduce systemic exposure and off‐target effects (see Table 1). Despite efforts to improve pharmacokinetic properties of ferritin‐based nanoparticles, it remains uncertain whether findings from murine models will translate effectively to humans.

### 2.3 Cellular Trafficking, Uptake, and Secretion

Knowledge of the intracellular trafficking and fate of ferritins is pivotal for their effective utilization as nanocarriers targeted toward specific sub‐cellular compartments. This facilitates the delivery of biomolecules such as contrast agents, genes, and drugs. The mode of entry and intracellular localization play significant roles in determining the induction of cytotoxicity. Therefore, comprehending cellular uptake and intracellular trafficking of nanoparticles is crucial for the development of safe and efficient nanomedicines.

**FIGURE 5:**
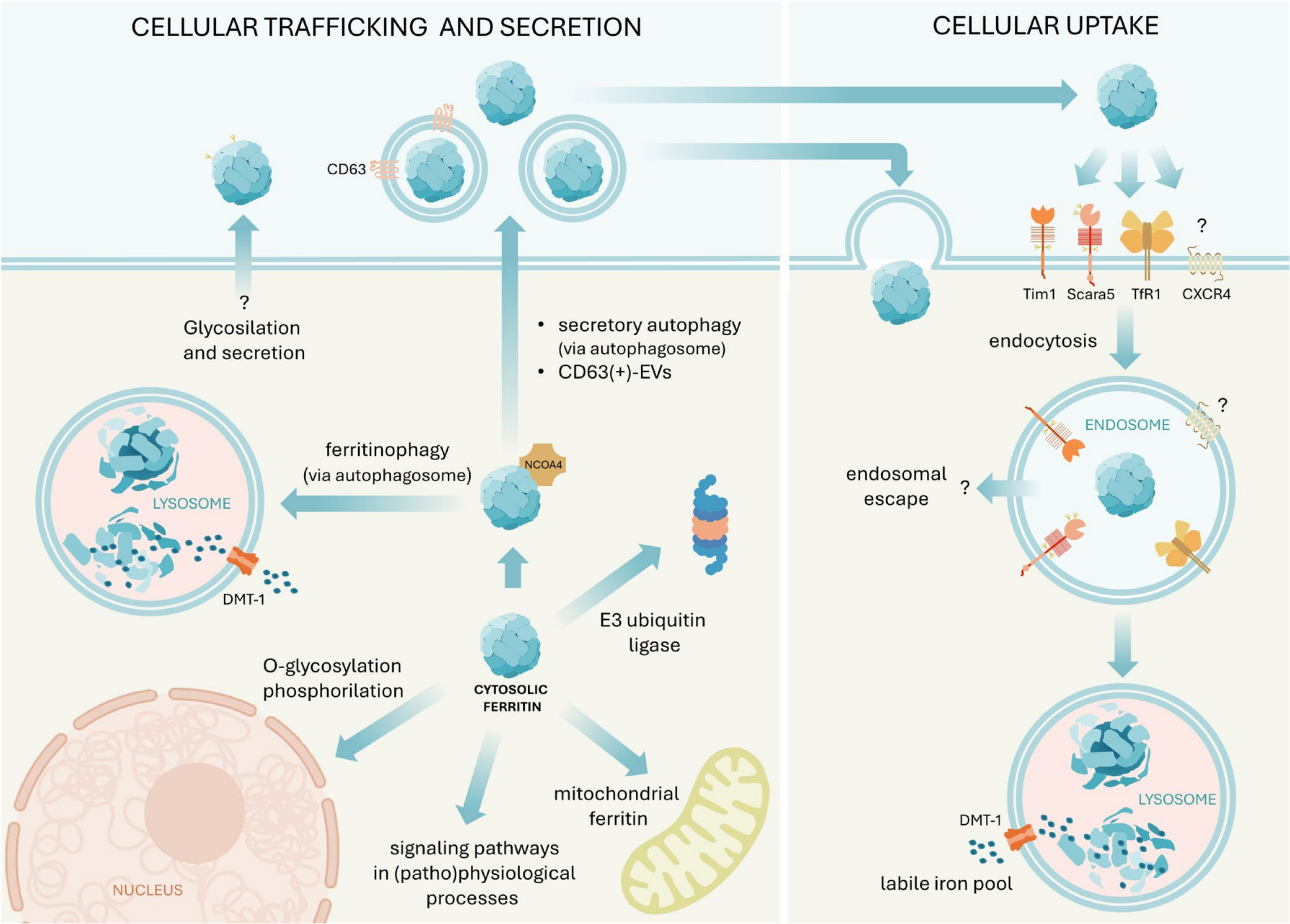
Cellular uptake, intracellular trafficking, and secretion of ferritin. Ferritin uptake by cells (right panel) occurs primarily through endocytosis following interaction with specific receptors such as CD71, Tim‐1, and SCARA5, while the uptake mechanism through CXCR4 remains unclear. Ferritin can also be internalized via extracellular vesicles (EVs). Once endocytosed, ferritin is typically degraded in the lysosome, releasing its iron content, although endosomal escape cannot be excluded. In the cytoplasm (left panel), ferritin is assembled and functions as an iron storage protein. It can be targeted to specific organelles, such as the nucleus and mitochondria, or be degraded by the proteasome. Ferritin also plays a role in intracellular signaling pathways. Interaction with cytosolic NCOA4 facilitates ferritinophagy in the lysosomes, where ferritin is degraded, and iron is released. Alternatively, ferritin can be secreted through secretory autophagy, being released in the form of vesicles, including CD63(+)‐EVs. The secretory pathway of glycosylated ferritin remains unclear, although it is known to involve macrophages and hepatocytes releasing partially glycosylated ferritin.

In physiological conditions, cellular ferritin originates from three distinct sources: (1) intracellular ferritin, synthesized within the cell's cytoplasm for iron storage, potentially destined for various cellular compartments such as the cytosol, nucleus, mitochondria, and lysosomes (Li et al. 2006; Surguladze et al. 2005; Levi et al. 2021; Asano et al. 2011); (2) extracellular ferritin, taken up by cells, characterized by a relatively low iron content, predominantly composed of L‐subunits with minimal H subunit presence, and glycosylated (50%–80%) (Ghosh, Hevi, and Chuck 2004); (3) extracellular ferritin present in exocytotic vesicles, exhibiting comparable iron levels to intracellular ferritin and maintaining the same L/H subunit ratio (Truman‐Rosentsvit et al. 2018; Figure 5).

Regarding endogenous cytoplasmic and mitochondrial ferritins, they follow complex intracellular trafficking routes to reach the designated destinations within the cell. Post‐translation, ferritin subunits assemble into the characteristic nanocage structure within the cytoplasm. Following assembly, ferritin may undergo targeting to specific organelles or secretory pathways for storage, secretion, or participation in intracellular signaling (Kotla et al. 2022; Figure 5). The regulation of ferritin trafficking ensures its appropriate distribution and involvement in various cellular processes.

As for serum ferritin, it is primarily secreted from macrophages during inflammatory conditions (Kannengiesser et al. 2009; Ghosh, Hevi, and Chuck 2004), although it may also originate from damaged cells (Kell and Pretorius 2014). Unlike cytosolic ferritin, serum ferritin is partially glycosylated and typically contains lower iron levels (Herbert et al. 1997). Serum ferritin is predominantly internalized via receptor‐mediated endocytosis, targeting various organs or tissues depending on the composition of H/L subunits, which in turn determines the recognition of specific receptors. H ferritin predominantly binds CD71 and Tim1 receptors (Chiou et al. 2018), whereas L ferritin, being the most abundant in serum, binds Scara5 (Mendes‐Jorge et al. 2014), which exhibits relatively broad tissue distribution and facilitates ferritin internalization for either ferritin elimination or iron delivery.

L ferritin also engages with other scavenger receptor members of class A (SR‐A), notably SCARA‐1 and MARCO, which are abundantly expressed in macrophages (Yu et al. 2020). Even though these receptors are not involved in the uptake processes, they have the potential to monitor ferritin levels and trigger inflammatory responses. Additionally, H ferritin interacts with both the C‐ and N‐terminal regions of the extracellular chemokine receptor CXCR4 (Li et al. 2006), which is implicated in lymphocyte migration and various physiological and pathological processes. CXCR4 is widely expressed in different cell types, including hematopoietic and endothelial cells, neurons, and stem cells. Moreover, elevated levels of CXCR4 are observed in cancer cells (Zlotnik 2006). While the interaction with the cytosolic C‐terminal region of CXCR4 leads to ferritin phosphorylation and subsequent nuclear migration, it remains unclear whether the interaction with the extracellular N‐terminal domain induces endocytosis (Li et al. 2006).

Although not all receptors are simultaneously required for ferritin endocytosis, they have all been identified within the endosome. The subsequent trafficking of ferritin after endocytosis remains incompletely understood. Various hypotheses suggest that ferritin may either be directed to the lysosome for protein degradation or exit the endosome through alternative pathways (Li et al. 2006; Chiou and Connor 2018; Figure 5). Moreover, ferritin can gain entry into cells through extracellular vesicles (Truman‐Rosentsvit et al. 2018; Ito et al. 2021; Gurung et al. 2021). The cellular trafficking of cytosolic ferritin is contingent upon the cellular iron concentration. When iron levels are low, ferritin synthesized in the cytosol is directed to lysosomes for degradation in response to iron deprivation. Iron stored within ferritin is then released back into the cytoplasm via ferritinophagy (Wang et al. 2023), a selective autophagy process. In ferritinophagy, iron‐loaded ferritin is trafficked to the lysosome where the protein shell is degraded, and the iron core is released and chelated by ascorbic acid and glutathione. The first committed step of ferritinophagy involves the binding of the nuclear receptor activator 4 (NCOA4) to the ferritin H subunits (Hoelzgen et al. 2024; Wang and Zhang 2022). This process leads to the formation of a ferritin‐NCOA4 condensate in the cytoplasm, which undergoes macroautophagy for lysosomal degradation or may be stored as hemosiderin via endosomal microautophagy.

Intracellular ferritins also encompass mitochondrial ferritin, which is encoded by a nuclear gene (Levi et al. 2021). This ferritin is synthesized as a precursor on cytosolic ribosomes, targeted to mitochondria, and ultimately processed into a functional protein involved in scavenging reactive oxygen species rather than iron storage. Another potential fate of intracellular ferritin is polyubiquitination and subsequent degradation by the proteasome (Voss et al. 2006).

Ferritin has also the capability to exit the cell via the secretory autophagy pathway, particularly activated in lysosome‐damaged cells (Kimura et al. 2017). In this process, the autophagosome merges with late endosomes, forming hybrid organelles termed amphisomes. Subsequently, the contents of amphisomes are exocytosed in the form of extracellular vesicles and particles (Solvik et al. 2022).

Upon iron replenishment, cellular iron levels are regulated by suppressing iron uptake and promoting iron excretion. These processes involve downregulating CD71 expression, increasing iron export via ferroportin, sequestering excess iron in ferritin, and secreting it through CD63(+) extracellular vesicles (EVs) (Torti and Torti 2021). These CD63(+) vesicles contain iron‐rich L and H ferritin (Yanatori, Kishi, et al. 2023; Yanatori, Nishina, et al. 2023). Iron loading facilitates the formation of holoferritin‐NCOA4 complexes, which are engulfed by endosomes and transported to CD63(+)‐EVs. Upon uptake into neighboring cells, these vesicles can induce iron overload and cellular damage (Toyokuni et al. 2021).

Furthermore, L ferritin can be secreted in a glycosylated form via the classical secretory pathway during translation, despite the absence of a conventional signal sequence (Ghosh, Hevi, and Chuck 2004). Under normal conditions, 50%–80% of serum ferritin is glycosylated due to its release from macrophages of the reticuloendothelial system and, to a lesser extent, hepatocytes (Sandnes et al. 2021). However, the details of this pathway are not yet fully understood.

Given the intricate nature of endogenously produced ferritin's intracellular trafficking, employing it as a nanoparticle for biomedical applications presents considerable challenges. External provision of ferritin raises questions about its potential impact on cellular metabolism and its resulting consequences. Typically, when ferritin is employed as a theranostic nanoparticle, the intended destination for the cargo is the cytosol or specific cellular compartments. However, upon endocytosis, ferritin primarily undergoes degradation at the endo‐lysosomal level. It remains unclear whether ferritin H or L subunits are capable of escaping from endosomes. Thus, a significant challenge in designing ferritin‐based nanoparticles lies in precisely directing them to the desired cellular sites for optimal efficacy. While lysosomal degradation may be desirable for cargos resistant to acidic pH, the inability to escape endosomes can be limiting in other cases. Despite its importance, few studies in the literature explore the mechanisms of endosomal escape, with most focusing solely on the phenotypic effects of cargo without elucidating the release process. Modifying ferritin appropriately is essential to ensure endosomal escape. Two proposed mechanisms for mediating this escape are the proton sponge theory and charge‐mediated membrane disruption. The proton sponge theory involves endosomal disruption due to osmotic swelling triggered by proton recruitment, leading to the protonation of nonexposed amino acid residues in response to the organelles' low pH. Alternatively, membrane disruption happens when cationic residues from the protein shell interact with the surface of the endosomal membrane, resulting in the formation of pores that induce disruption (Stewart, Langer, and Jensen 2018). Several studies have demonstrated the efficacy of these approaches. For instance, modifying ferritin at the N‐terminal with a histidine sequence facilitated endosomal escape and cytosolic localization (Zhao et al. 2023). Another study showed that loading ferritin with a polyamine dendrimer binding a small nucleic acid led to cytosolic release and cargo processing, indicating that the positive charge of the dendrimer facilitated release via the proton sponge effect (Palombarini et al. 2021).

The destiny of ferritin upon its release from the endosome into the cytosol remains uncertain, as well as its potential impact on cellular metabolism. Furthermore, it remains unclear whether exogenously administered ferritin, upon cellular uptake, can be subsequently secreted with or without its payload. This aspect requires further investigation due to the expanding range of functions attributed to extracellular ferritin in contemporary research. These functions encompass various roles, such as iron delivery, immunity, inflammation, signaling, and cancer (Wang et al. 2010). These roles appear to be independent of the iron content within the ferritin molecule, indicating that the protein shell alone might function autonomously from its traditional role as an iron storage protein. For instance, H ferritin has been identified as a pro‐inflammatory signal in the liver, operating independently of its iron content through the activation of the nuclear factor NF‐kB pathway (Ruddell et al. 2009). Another study demonstrated that the induction of H apoferritin in response to polymicrobial infection is crucial for establishing disease tolerance to sepsis (Weis et al. 2017). Exogenous apoferritin has also been implicated in influencing critical processes of glutamatergic neurotransmission, potentially leading to neurological consequences. It was observed that exogenous ferritin in nerve terminals elevated ambient glutamate levels, reduced transporter‐mediated glutamate uptake, and decreased the proton electrochemical gradient of synaptic vesicles (Krisanova et al. 2014).

Moreover, owing to its iron‐binding capacity, apoferritin may participate in chelating excess iron, thus shielding cells from oxidative stress. It could potentially be involved in safeguarding neurons against Parkinson's disease (Foley, Hare, and Double 2022). However, excessive iron sequestration by administered apoferritin could lead to iron deficiency in certain tissues or disrupt normal iron homeostasis, particularly if the treatment regimen is not carefully monitored.

Given the various aspects of ferritin's cellular trafficking and its significant impact on cellular metabolism, it is crucial to comprehensively assess the influence of the ferritin protein shell. This evaluation should be carried out irrespective of the specific therapeutic drugs that might be loaded inside, as understanding the intrinsic properties and interactions of the protein shell can provide deeper insights into its overall effect on cellular functions.

### 2.4 Immunological Concerns

The utilization of ferritin nanoparticles as drug delivery agents presents several immunological challenges that require careful consideration. A prominent concern with biotherapeutic drugs and biological carriers, including ferritin nanoparticles, is the risk of immune reactions. Immunotoxicity is particularly worrisome, especially with nonhuman or recombinant ferritins. The immune system recognizes nanoparticles based on their surface and compositional characteristics, potentially triggering immune responses (Aljabali et al. 2023). Upon encountering biofluids, nanoparticles can form a “protein corona,” a layer of proteins that adsorbs onto the nanoparticle surface, which can significantly alter their bioactivity and interaction with the immune system. This protein corona can either mask or reveal specific epitopes, thereby influencing how the immune system perceives and reacts to the nanoparticles (Panico et al. 2022).

Phagocytes, including macrophages and dendritic cells, play a key role in the initial immune response to nanoparticles, potentially leading to immunostimulation or immunosuppression. This can result in inflammatory or autoimmune conditions (Ray et al. 2021). Although human H ferritin is a naturally occurring protein, its recombinant production can provoke immune responses due to possible structural changes during production, purification, and formulation processes, which exposes hidden residues and epitopes. Additionally, contaminants such as endotoxins from the production systems pose significant risks for unintentional immune responses. The risk of these immune responses is further amplified when using nonhuman ferritins or genetically/chemically modified human ferritins, which alter their structure and charge, potentially increasing their immunogenicity.

Research on the immune response to ferritin‐based nanoparticles is limited, with the majority of studies being conducted either in vitro (Sitia et al. 2023; Ravishankar et al. 2023) or using murine models (Jia et al. 2022; Chen et al. 2022). For example, a study using human whole blood samples to assess proinflammatory responses to human H ferritin nanocages found that immunogenicity could be triggered by pyrogenic contaminants (Sitia et al. 2023). Even after these contaminants were removed, a mild immunoreactivity persisted. However, further purification, involving the loading of antitumor drugs through standardized methods, eliminated the release of proinflammatory cytokines like IFN‐γ, IL‐6, and TNF‐α. This emphasizes the need to assess both acute toxicity and immunoreactivity when evaluating the safety of new nanodrugs for clinical use.

Another study investigated the immune response to bacterial ferritin nanoparticles in murine macrophages, showing significant upregulation of immune markers such as CCR2, IL1β, TNFα, and VCAM‐1, along with increased reactive oxygen species production (Ravishankar et al. 2023). The pronounced IL1β response highlighted the expected proinflammatory reaction to a bacterial protein. While in vitro studies provide valuable insights, they do not fully replicate the complexity of in vivo conditions or the intricacies of human organoid models (Sevieri et al. 2024). This discrepancy leads to variations in nanoparticle distribution, cellular uptake, and immune clearance observed between in vitro and in vivo systems. Dynamic flow models, designed to better mimic in vivo conditions, have demonstrated reduced nanoparticle toxicity compared to static cultures, highlighting the importance of physiological flow in these studies. In vivo studies using murine models are widely used to evaluate the immunotoxicity of nanoparticles. However, research specifically involving human ferritin nanoparticles in these models remains limited. These studies provide only partial insights because, despite the high homology between human and murine ferritin, there is still a possibility that mice will mount an immune response against what they perceive as a nonself protein. Murine models often fail to fully replicate human immune responses due to fundamental genetic differences, variations in molecular interactions, and differences in immune system maturity between mice and humans (Mak, Evaniew, and Ghert 2014). This makes it challenging to directly translate findings from mice to human clinical outcomes. For instance, specific pathogen‐free mice, which are frequently used in laboratory settings, possess immature immune systems that do not accurately reflect the immune function of adult humans (Reese et al. 2016; Beura et al. 2016). To overcome these limitations, humanized mouse models have been developed. These models incorporate human tumors and immune systems, providing more predictive data regarding human responses to nanoparticle‐based therapies. Nevertheless, even these advanced models present limitations, as they cannot fully replicate the complexity of human immune responses (De La Rochere et al. 2018).

Insights can also be drawn from conditions like hyperferritinemic syndrome, which may help understand the potential effects of human H ferritin‐based nanoparticles. This syndrome involves elevated serum ferritin levels and intense inflammation, leading to a cytokine storm characterized by elevated IL‐1β, IL‐6, IL‐10, IFN‐γ, TNF‐α, and other cytokines, potentially mimicking what might occur after administering ferritin‐based nanoformulations (Jia et al. 2022).

Currently, human experimental data on ferritin‐based systems are quite limited, primarily stemming from phase 1 clinical trials (NCT04784767, NCT03186781, NCT04579250, NCT03814720). These trials have primarily focused on ferritin's use as vaccine scaffold, rather than as a drug delivery vehicle. In these trials, *Helicobacter pylori* ferritin has been widely used and has effectively stimulated the production of specific antibodies. One exception (Houser et al. 2022) involved a vaccine using human ferritin to present influenza antigens. This study found no antibodies against the human ferritin shell, suggesting it lacks intrinsic immunogenicity in its natural form. However, modifications to its surface for targeted drug delivery could introduce new immunogenic properties, warranting further investigation. Overall, while current research has made significant strides in understanding the immune response to ferritin‐based nanoparticles, there remains a critical need for more advanced models that can accurately mimic human physiological conditions. Such advancements are essential for the reliable prediction of nanoparticle behavior and immune interactions in human clinical settings, ensuring their safe and effective application in medicine.

## 3 Conclusion

In this review, we aimed to provide a comprehensive overview of the physiological roles of ferritin and how these could be modulated by the use of exogenous human H ferritin in nanomedicine.

Despite numerous studies explored ferritin‐based nanoparticles for theranostic applications and vaccine development, there is still no established clinical use for these technologies. This limitation is not unique to ferritin but is also observed with nanoparticles of various types. Ferritin offers several advantages over non‐biological nanoparticles, such as high biocompatibility due to its status as a physiological protein. However, this inherent biocompatibility can be a double‐edged sword as formulating human H ferritin as a nanoparticle may disrupt cellular metabolic balance. Ferritin's critical involvement in iron homeostasis and its roles in infection response, inflammation, and immunomodulation mean that exogenous ferritin could potentially perturb these processes, leading to undesirable effects.

Many studies have demonstrated the efficacy of ferritin nanoparticles in active targeting for theranostic applications. However, these studies often overlook the potential impacts of the ferritin protein shell on cellular biochemistry and physiology independently of the therapeutic payload. This oversight could be a significant barrier to clinical translation, as adverse effects might be mediated not only by the payload but also by the protein shell itself.

Another major challenge is biodistribution. Most research has been conducted using murine models, which have ferritin receptors that differ from those in humans, complicating the translation of these findings to clinical settings. Although ferritin tends to accumulate in tumors due to its active and passive targeting properties, it also distributes to other organs, such as the liver, spleen, and kidneys. This nonspecific biodistribution raises concerns about off‐target effects and potential toxicity.

To facilitate the clinical application of human H ferritin‐based nanoparticles, it is crucial to design particles with an extended half‐life in circulation and optimized biodistribution. This could be achieved by engineering ferritin to target specific cell types or tissues while minimizing recognition by CD71, a receptor overexpressed in tumor tissues but also present in many healthy, metabolically active tissues. Alternatively, exploiting the natural biodistribution of ferritin to target organs where it naturally accumulates, such as the liver, could be beneficial for treating noncancerous conditions. It is worth noting that research on using nanoparticles for noncancerous conditions is limited, despite these conditions being responsible for a significant number of deaths globally.

In conclusion, while human H ferritin‐based nanoparticles hold promise for various medical applications, several critical challenges must be addressed to transition from research to clinical practice. These include achieving selective targeting, understanding and controlling biodistribution, and comprehensively elucidating the impact of exogenous human H ferritin protein shell on cellular biochemistry. Addressing these challenges will be essential for the successful clinical translation of ferritin‐based nanomedicine.

## Author Contributions

**Alberto Macone:** conceptualization (equal), data curation (equal), funding acquisition (equal), software (equal), supervision (equal), visualization (equal), writing – original draft (lead), writing – review and editing (lead). **Chiara Cappelletti:** investigation (equal), visualization (supporting), writing – original draft (supporting). **Alessio Incocciati:** investigation (equal), resources (equal), writing – original draft (supporting), writing – review and editing (supporting). **Roberta Piacentini:** investigation (equal), resources (equal), writing – original draft (supporting), writing – review and editing (supporting). **Sofia Botta:** investigation (equal), methodology (equal), writing – original draft (supporting), writing – review and editing (supporting). **Alberto Boffi:** project administration (equal), writing – review and editing (equal). **Alessandra Bonamore:** conceptualization (equal), data curation (equal), funding acquisition (equal), resources (equal), software (equal), supervision (equal), writing – original draft (lead), writing – review and editing (lead).

## Conflicts of Interest

The authors declare no conflicts of interest.

## Related WIREs Articles

Human ferritin for tumor detection and therapy

The development of nanocarriers for natural products

## Data Availability

Not applicable.
